# Exposure–Response Functions for the Effects of Traffic Noise on Self-Reported Annoyance and Sleep Disturbance in Finland: Effect of Exposure Estimation Method

**DOI:** 10.3390/ijerph19031314

**Published:** 2022-01-25

**Authors:** Tarja Yli-Tuomi, Anu W. Turunen, Pekka Tiittanen, Timo Lanki

**Affiliations:** 1 Finnish Institute for Health and Welfare, P.O. Box 95, FI-70100 Kuopio, Finland; anu.turunen@thl.fi (A.W.T.); pekka.tiittanen@thl.fi (P.T.); timo.lanki@thl.fi (T.L.); 2 Department of Environmental and Biological Sciences, University of Eastern Finland, P.O. Box 1627, FI-70211 Kuopio, Finland; 3 School of Medicine, University of Eastern Finland, P.O. Box 1627, FI-70211 Kuopio, Finland

**Keywords:** traffic noise, annoyance, sleep disturbance, exposure–response relationship

## Abstract

Large variations in transportation noise tolerance have been reported between communities. In addition to population sensitivity, exposure–response functions (ERFs) for the effects of transportation noise depend on the exposure estimation method used. In the EU, the new CNOSSOS-EU method will change the estimations of exposure by changing the assignment of noise levels and populations to buildings. This method was officially used for the first time in the strategic noise mapping performed by Finnish authorities in 2017. Compared to the old method, the number of people exposed to traffic noise above 55 dB decreased by 50%. The main aim of this study, conducted in the Helsinki Capital Region, Finland, was to evaluate how the exposure estimation method affects ERFs for road traffic noise. As an example, with a façade road traffic noise level of 65 dB, the ERF based on the highest façade noise level of the residential building resulted in 5.1% being highly annoyed (HA_V_), while the ERF based on the exposure estimation method that is similar to the CNOSSOS-EU method resulted in 13.6%. Thus, the substantial increase in the health effect estimate compensates for the reduction in the number of highly exposed people. This demonstrates the need for purpose–fitted ERFs when the CNOSSOS-EU method is used to estimate exposure in the health impact assessment of transportation noise.

## 1. Introduction

Based on the noise reporting survey performed in the EU in 2017, more than 112 million inhabitants were exposed to average day–evening–night road traffic-related noise levels (L_den_) ≥ 55 dB, and more than 78 million to night noise levels (L_night_) ≥ 50 dB, in EEA-33 countries [1]. The corresponding numbers for rail traffic were 21 and 17 million. L_den_ 55 dB and L_night_ 50 dB are the EU thresholds for excess exposure, as defined in the Environmental Noise Directive (END). Based on burden of disease (BoD) assessment, traffic noise is one of the most important environmental risks to human health in Europe [2]. In 2011, the World Health Organization (WHO) Europe estimated that the main health burdens related to traffic noise resulted from sleep disturbance and annoyance [3]. 

BoD calculations are based on the number of exposed individuals and exposure–response functions (ERFs). In environmental epidemiology, ERF is used to quantify population responses, such as annoyance or health effect, at different levels of exposure to a certain environmental stressor. ERFs generally depend on the exposure time. In this paper, the effects of long-term noise exposure are considered.

The most recent reviews and analyses of ERFs for traffic noise are presented in WHO’s systematic reviews on environmental noise and annoyance [4] and sleep disturbance [5]. These analyses include meta-analyses of several national surveys. Based on the reviews, there is high variation between the studies in terms of the methodology used in the social surveys and the exposure estimation, as well as between the communities (for example, population, building stock, habits, and meteorology). As a result of these differences, the ERFs fitted to the data (observed health effect vs. exposure) typically explain less than 40% of the variance, and the prediction interval is very large [6].

Fidell et al. [7] and Schomer et al. [8] introduced a theory-based approach to predict the prevalence of annoyance. They defined the community tolerance level (L_ct_) as a day–night sound level at which 50% of the people in a particular community are predicted to be highly annoyed. L_ct_ accounts for differences between sources and/or communities when predicting the percentage of those highly annoyed by noise. This theory-based method is included in the 2016 revision of ISO 1996-1 [6].

The ERFs likely depend, at least to some extent, on the method used for exposure estimation. Unfortunately, the exposure estimation method is not always described in detail in publications. This leads to higher uncertainty in generalized ERFs based on meta- or re-analyses of several original studies. 

In the END calculations, countries have used different methodologies to assign their populations to dwellings. In 2012, Kephalopoulos et al. [9] published a methodological framework for Common Noise Assessment Methods in Europe (CNOSSOS-EU). They proposed a revised definition, under which the most exposed façade is the external wall of the dwelling exposed to the highest value of L_den_ or L_night_ from the specific noise source under consideration. For buildings containing multiple dwellings, when the specific layout of said dwellings is not known, the approach is based on the equal distribution principle, whereby residents are assigned equally to all façade noise receivers (i.e., locations where predictions of noise levels are available). Previously, the most exposed façade was determined as “the external wall facing onto and nearest to the specific noise source”. Thus, it was not defined whether it was the most exposed façade of the building or a dwelling. For example, in Finland, the locations of dwellings within a building are not known, and previously all inhabitants have been assigned to the building’s highest façade noise level [10]. 

The CNOSSOS-EU method was established in the Commission Directive (EU) 2015/996 of 19 May 2015. In Finland, the CNOSSOS-EU method was already being used in the 2017 round. In the Helsinki Capital Region, the numbers of people exposed to high traffic noise levels were compared between the old (all inhabitants assigned to the highest façade noise level of the building) and the new (equal distribution) methods [10]. The results for Helsinki are shown in Table 1. Using the new method, the number of residents exposed to such noise was, on average, less than half that given by the old calculation method. 

During the next round of END in 2022, the CNOSSOS-EU method will be used in all countries. This will remarkably decrease the estimated number of people exposed to high traffic noise levels. The equal distribution principle leads to more precise exposure estimation, and thus the old exposure–response functions may become unfit. 

The aim of this study was to evaluate the effect of the exposure assessment approach on the exposure–response functions. In addition, ERFs for the effects of road and rail traffic noise on annoyance and sleep disturbance are provided for cities in subarctic climate regions, where the thermal insulation of residential buildings also enhances the sound insulation.

## 2. Materials and Methods

### 2.1. Study Design and Participants

The Helsinki Capital Region Environmental Health Survey was conducted in Helsinki, Espoo and Vantaa, in Finland. The survey was carried out to evaluate residents’ perceived exposures to specific environmental factors and their views of health risks caused by the environment. It aimed at assessing the health risks associated with noise and air pollution exposure and the potential health benefits of access to green areas. Additionally, some history of medical conditions and data on confounders were collected to facilitate epidemiological analysis. The questionnaire had 93 questions and numerous sub-questions. The survey was conducted in two phases: firstly, in the city of Helsinki from the latter part of May to August of 2015; secondly, in the cities of Espoo and Vantaa from June to August of 2016. In total, 8000 residents aged 25 years and above were chosen from the Population Registry of Finland using simple random sampling in 2015; the same number of residents was sampled in 2016, yielding a total sample of 16,000 residents. Potential respondents were contacted by post and invited to fill in a self-administered questionnaire, which they could choose to complete on paper or electronically. One reminder was sent to non-respondents. This procedure resulted in 7321 valid records.

### 2.2. Annoyance and Sleep Disturbance 

Subjective noise annoyance was assessed using the questionnaire item, “Are you usually disturbed by road traffic noise (i.e., noise annoys you, disturbs your concentration, etc.) indoors at home when the windows are closed?” The response options were: (1) not at all; (2) slightly; (3) moderately; (4) very; (5) extremely. This question deviates from the instructions of ISO/TS 15666:2003 [11]. The location (home indoors) and window position (closed) were added because in Finland the “Decree of the Ministry of Social Affairs and Health on Health-related Conditions of Housing and Other Residential Buildings and Qualification Requirements for Third-party Experts” requires these circumstances when estimating disturbance caused by noise at home [12]. 

Subjective sleep disturbance was assessed using the questionnaire item, “Is your sleep usually disturbed by road traffic noise (i.e., noise prevents you from falling asleep, wakes you up) at home?” The response options to this question were: (1) not at all; (2) slightly; (3) moderately; (4) very; (5) extremely. The same questions about annoyance and sleep disturbance were then asked separately for rail traffic noise.

### 2.3. Window Orientation

The following questions were asked to find out the orientations of the windows in respondent’s dwellings (response options in parenthesis): “In which direction your dwelling windows are facing? ((1) the street; (2) the yard; (3) both the street and the yard)”; “In which direction your bedroom windows are facing? ((1) the street; (2) the yard)”. 

### 2.4. Exposure Estimation

L_den_ and L_night_ were used as the exposure indicators. L_den_ is a descriptor of A-weighted noise level based on long-term energy equivalent noise level (L_eq_) over a whole day, with a penalty of 5 dB for evening noise (19.00–22.00) and 10 dB for nighttime noise (22.00–7.00) to reflect people’s extra sensitivity to noise during evening and night. L_night_ is the A-weighted long-term average sound level for nighttime (22.00–7.00). 

The residential exposure to road and rail traffic noise was estimated from façade noise maps, which were modeled for the Helsinki Capital Region by the Sito consulting company. Sito carried out the 2017 noise calculations in accordance with the Environmental Noise Directive 2002/49/EC, using the Common Noise Assessment Methods in Europe (CNOSSOS-EU; the Commission Directive (EU) 2015/996 of 19 May 2015) for road and rail traffic noise. The noise models were based on 2016 data. There have been no major changes in the traffic situation in the years prior to the survey, and thus, the noise conditions have remained stable in the study area.

**Figure 1:**
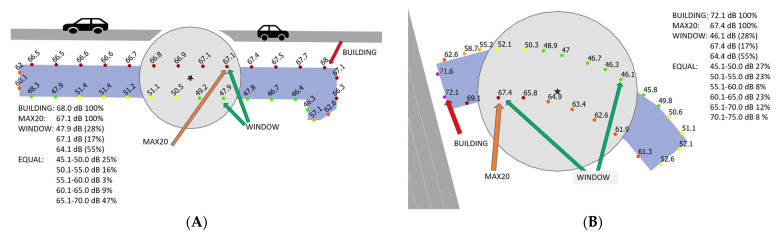
Examples of exposure estimates when the building is parallel (**A**) and perpendicular (**B**) to the road. The distributions of residents exposed to different noise levels are presented for the methods used in this paper and for the equal distribution method.

In order to study the effects of using different exposure assessment methods on exposure–response functions, three sets of L_den_ and L_night_ were used in this study for road traffic noise: (1) BUILDING: The highest façade noise level of the residential building was used for all respondents living in that building; (2) MAX20: the highest L_den_ or L_night_ at the façade points within 20 m of the center point of the building, as registered by the Digital and Population Data Services Agency for the resident’s home address, were used; (3) WINDOW: Depending on the directions of all windows in the dwelling (annoyance assessment) or the bedroom window (sleep disturbance), the highest (window(s) facing street), the lowest (window(s) facing yard), or the logarithmic average of the highest and lowest (windows facing both the street and the yard) L_den_ or L_night_ at the façade points within 20 m of resident’s home address were used in the exposure assessment. In Finland, the BUILDING method was used during the 1st and 2nd rounds of strategic noise mapping, because no data are available on dwelling location and the distribution of residents within the building [10]. MAX20 is the best estimate for the average highest façade noise level in all dwellings in a given building when the window directions are not known, while the WINDOW method gives the best exposure estimate if window directions are known but there are no data on dwelling location within the building. Examples of the exposure assessment methods are shown in Figure 1 in the Results. For rail traffic noise only the MAX20 method was available. 

### 2.5. Outcome Variables

A special outcome variable is the percentage of “highly annoyed” study participants. Those who are highly annoyed are individuals that respond in the upper parts of the annoyance response scale. Based on the recommendation of the ICBEN team’s “community response to noise” [13], the top two points of the 5-point scale (“very” and “extremely”) were combined to measure the percentage of highly annoyed and will be referred to as HA_V_ [11]. The same 60% cut-off point was used to estimate the “percentage of highly sleep disturbed” (HSDV) respondents. 

### 2.6. Exposure–Response Functions

For road traffic noise, L_den_ and L_night_ were divided into intervals of 1 dB. If such an interval contained less than 100 cases, it was combined with the adjacent interval with the least observations. This step was repeated until every interval contained at least 100 cases. This procedure was modified from [14]. For each resulting interval, the midpoint and the outcome variables were determined and plotted. For rail noise, intervals of 5 dB were used because the number of exposed individuals was much smaller, especially at the high noise levels. The exposure–response functions were only specified for L_den_ ≥ 45 dB and L_night_ ≥ 40 dB. Low exposure levels were excluded from the analyses because, in general, the assessment of those levels is relatively inaccurate, and when noise levels are low, other noise sources than traffic may be more significant.

Community tolerance level (L_ct_den_) was determined for the study population. L_ct_ is defined as the day–night sound level (L_dn_) at which 50% of the people in a particular community are predicted to be highly annoyed by noise exposure [6,7,8]. L_ct_ is used as a parameter that accounts for differences between sources and/or communities when predicting the percentage of highly annoyed by noise exposure. A community with a higher L_ct_ is more tolerant to noise than a community with a lower L_ct_. In addition to community properties, L_ct_ also depends on the exposure estimation method. In this paper, L_ct_den_ is defined as the L_den_ at which HA_V_ = 50 and is determined empirically by finding the value of L_ct_den_ (to a precision of 0.1 dB) that minimizes the root mean square difference between the theory-based equation (Equation (1)) and pairs of empirical (L_den_, HA_V_) observations derived from the questionnaire study. This procedure was modified from [6].

HA_V_ = 100 EXP(−(1/(10^((L_den_ − L_ct_den_ + 5.306)/10))^0.3)) (1)

There is no theory-based equation available for the HSD_V_ in the literature. We modified Equation (1) to give the best fit for HSD_V_ in our data and determined L_ct_night_ as the L_night_ at which HSD_V_ = 50. Only observations with 1 dB intervals were used in the determination of L_ct_den_ and L_ct_night_.

ERFs were also determined using regression formulation. However, the coefficients of determination (R^2^) between the exposure and the outcome variable were lower for 2nd or 3rd degree polynomial functions (best fit) than for Equation (1), and thus these functions are not shown.

It should be noted that all the ERFs presented in this paper are only applicable to existing situations and long-term (annual) averages of traffic noise. They should not be used with shorter time periods, such as weekends or during a brief increase in road traffic. In newly created situations, especially when the community is not familiar with the sound source in question, higher community annoyance can be expected. This difference may be up to 5 dB [6].

## 3. Results

### 3.1. Road Traffic Noise

Overall, 7321 valid records were obtained from the study participants, giving a response rate of about 50% for women and 41% for men. The modeled façade road traffic noise level was available for 7138 respondents. Responses to the question concerning annoyance and/or sleep disturbance due to road traffic noise were missing for 191 participants. Information about the window direction of the dwelling and/or bedroom was contradictory for 78 and missing for 115 participants. The characteristics of the study groups that are valid for use in the determination of the exposure–response function are given in Table 2. In multi-story residential buildings, 17% of the respondents had access only to the most exposed façade, while 28% had all their windows facing the yard. Information about respondents with MAX20 L_den_ < 45 dB and L_night_ < 40 dB is given in Table S1 in the Supplementary Materials.

In Figure 1, examples of the exposure assessment methods are shown, and the distributions of residents exposed to different noise levels are compared with the equal distribution method used in the CNOSSOS-EU. The distribution of residents employed in the WINDOW method is based on the questionnaire results of respondents living in multi-story residential buildings. For rail traffic noise, only MAX20 method was available. 

**Figure 2:**
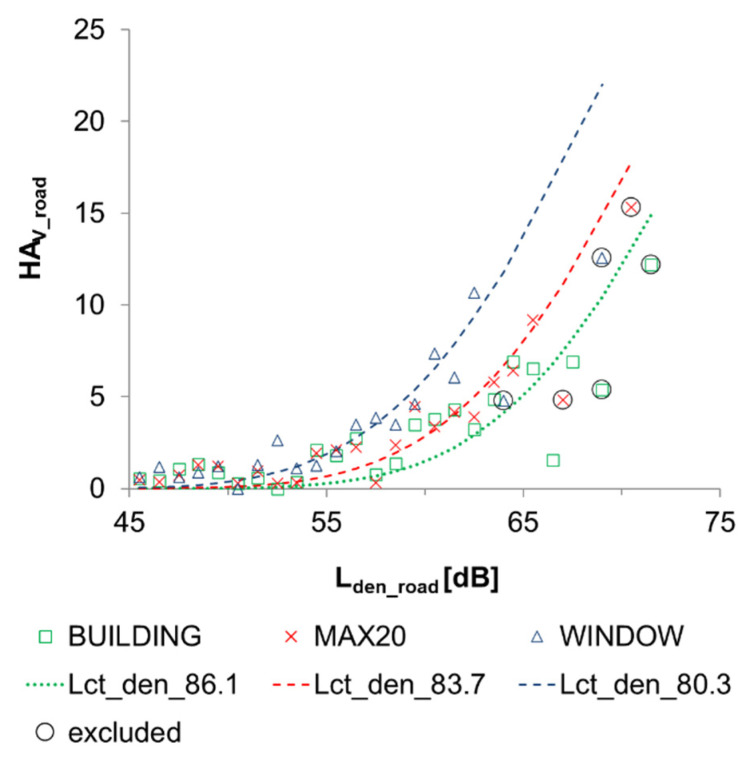
The percentage of highly annoyed (HA_v_) respondents due to road traffic noise inside their home at different L_den_ levels outside of the façade. Green squares present the observations when L_den_ equals the highest façade point level of the residential building; red crosses describe the situation where the exposure estimate is the highest façade point level within 20 m from the center point of the building; blue triangles describe the situation where access to the quiet façade has been taken into consideration in the exposure assessment. L_ct_den_ lines show community tolerance levels that best fit Equation (1) to the empirical observations. Observations for intervals wider than 1 dB have been circled and excluded from the L_ct_den_ fitting.

The results for the community tolerance level L_ct_den_ are given in Table 3. Figure 2 shows graphs of Equation (1), using these L_ct_den_ values as well as the empirical (L_den_, HA_V_) observations derived from the questionnaire study. The observations for intervals wider than 1 dB resulted in considerably lower HAv-estimates than the 1 dB data and were thus excluded from the L_ct_den_ fitting.

The best fit for road traffic L_night_ and HSD_v_ was observed with the following modification of Equation (1):

HSD_V_ = 100 EXP(−(1/(10^((L_night_ − L_ct_night_ + 8.0)/10))^0.2)) (2)

**Figure 3:**
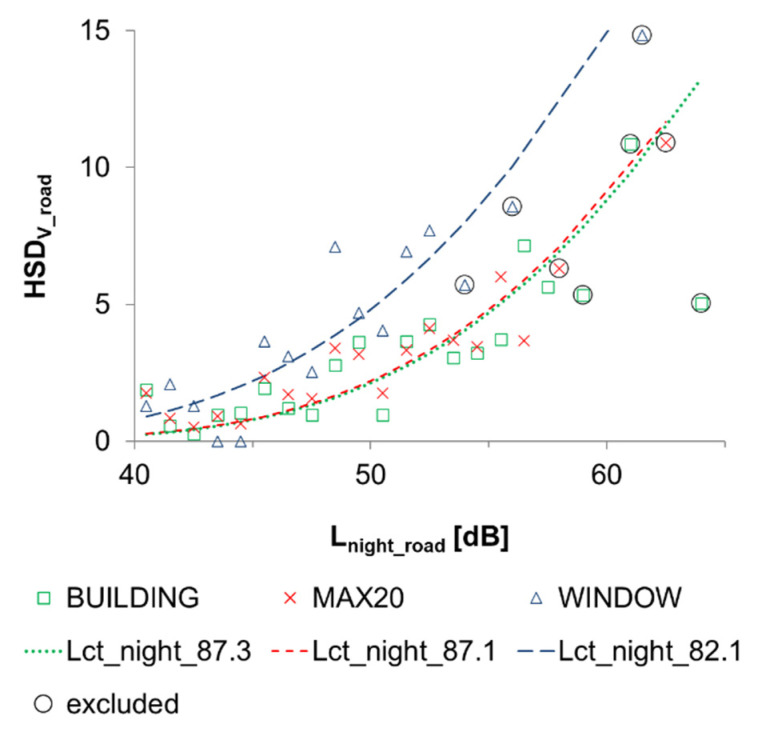
The percentage of highly sleep disturbed (HSD_V_) respondents due to road traffic noise inside their home at different L_night_ levels outside of the façade. Green squares represent the observations made when L_night_ equals the highest façade point level of the residential building; red crosses describe the situation where the exposure estimate is the highest façade point level within 20 m from the center point of the building; blue triangles describe the situation where access to the quiet façade has been taken into consideration in the exposure assessment. L_ct_night_ lines show community tolerance levels that best fit Equation (2) to the empirical observations. Observations for intervals wider than 1 dB have been circled and excluded from the L_ct_night_ fitting.

The power was decreased from 0.3 to 0.2, which gives a more gradual slope to the curve. Consequently, the constant was changed from 5.3 to 8.0 in order to retain L_ct_night_ as the 50% point. The results for the community tolerance level L_ct_night_ are given in Table 3 and Figure 3.

### 3.2. Rail Traffic Noise

**Figure 4:**
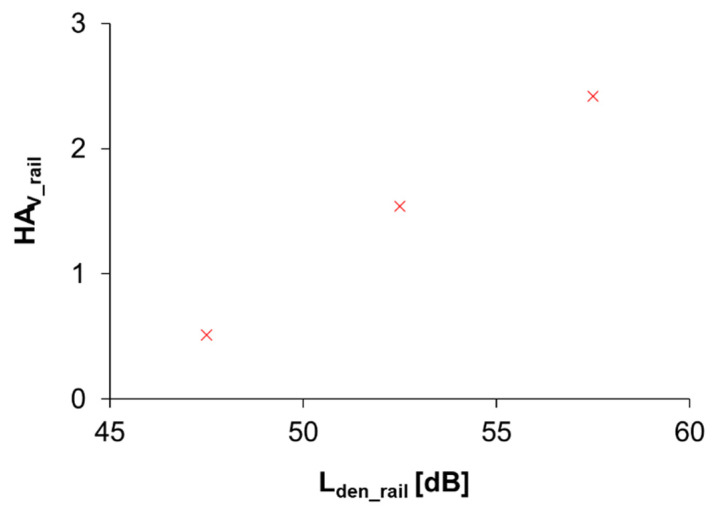
The HA_V_ respondents related to rail traffic noise inside their home at different L_den_ levels outside of the façade. Exposure was estimated as the highest façade point noise level within 20 m from the home coordinates.

Modeled building façade rail traffic noise level was available for 3344 respondents. Responses to the question concerning annoyance due to rail traffic noise were missing from 8 participants; responses to the sleep disturbance question were missing from 36, and responses to both annoyance and sleep disturbance questions were missing from 5 participants. Information about window orientation in relation to the railway was not available. The characteristics of the study groups that are valid for use in the determination of the ERFs are given in Table 2. Only 66 respondents were exposed to L_den_ 60 dB or higher. ERF for rail traffic noise and HA_V_ could not be estimated based on data from three 5 dB intervals. Observations for below 60 dB are shown in Figure 4.

Data for HSD_V_ were even more sparse than for HA_V_. Only 5 respondents were exposed to rail traffic noise L_night_ 60 dB or higher, and only 9 respondents out of 3303 were highly sleep disturbed because of rail traffic noise. For L_night_ 45–50 dB, the HSD_v_ was 2. 

### 3.3. Comparison

**Figure 5:**
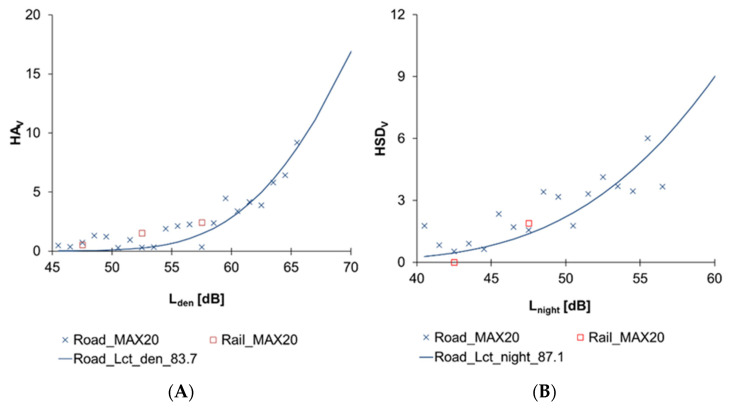
Comparison of (**A**) HAV and (**B**) HSDV between road and rail traffic noise.

The HA_V_ and HSD_V_ for road and rail traffic noise are presented in Figure 5A,B. The highest L_den_ and L_night_ at façade points within 20 m of the home coordinates have been used as the exposure estimates. Based on visual observation, no difference was found between road and rail traffic. 

### 3.4. Combination of Population Assignment Method and ERF

In Finland, the 2017 END calculations were carried out using the CNOSSOS-EU method, and the number of residents exposed to L_den_ above 55 dB was generally less than half of the number given by the old calculation method (Table 1). The HA_v_ and HSD_v_ for Helsinki were calculated using the number of residents exposed from Table 1 and the ERFs based on L_CT_den_ and L_CT_night_ (Table 3). Different combinations of exposure estimation methods (BUILDING or EQUAL) and ERFs (BUILDING or WINDOW) were used. The results in Table 4 show that a wrong combination (when the exposure assessment method for ERF and the impact assessment are not compatible) would lead to a remarkable decrease in both HAv and HSDv.

## 4. Discussion

The exposure–response functions between road traffic noise and the percentage of highly annoyed and highly sleep disturbed respondents in the Helsinki Capital Region Environmental Health Survey were studied using three different exposure assessment methods. In addition, HA_V_ was calculated for rail traffic noise using the highest façade noise level within 20 m of the coordinates of the dwelling. 

There are inaccuracies in all the exposure assessment methods currently available for population level studies on the health effects of noise, including the ones used in this study. Home indoor noise levels would give more accurate exposure estimates, but the values of the variables affecting the sound insulation of dwellings are typically unknown. In this study, individuals reported their annoyance and sleep disturbance at the noise level that they were actually exposed to when at home, indoors, with the windows closed. Therefore, the noise exposure estimate, that is, the noise level modeled at the façade of their dwelling, was much higher than the actual exposure level. The magnitude of this error depends on the exposure assessment method selected. Under the BUILDING and MAX20 methods, the noise level at the most exposed façade of the dwelling is likely to be overestimated, especially for buildings containing several dwellings, and thus annoyance at certain façade noise levels may be misclassified into a higher noise class. This was observed in the results, as the HA_V_ and HSD_V_ decreased from WINDOW to MAX20, and again to BUILDING.

The data on the higher levels of rail traffic noise were, unfortunately, too sparse for use in the determination of exposure–response functions. The empirical observations derived from the questionnaire study were close to those for road traffic noise, but the effects of the different intervals used for the calculations (1 dB for road and 5–10 dB for rail traffic) are unclear. In the road traffic data, intervals above 1 dB tended to produce lower responses and were thus excluded from the analysis. 

In multi-story residential buildings, 17% of the respondents only had access to the most exposed façade of the building, and 28%, only to the yard. Thus, using the CNOSSOS-EU method for assigning noise levels and populations to buildings gives more accurate estimates for the number of citizens in each noise class than the old method, under which the highest façade noise level of the entire residential building was allocated to all residents living in that building. In Finland, the 2017 END calculations were carried out using the CNOSSOS-EU method, and the number of residents exposed to L_den_ above 55 dB was generally less than half that given by the old calculation method. However, the ERF defined for the old exposure assessment method cannot be easily accommodated with the CNOSSOS-EU method. Based on our results, the estimates of adverse health effects will not decrease accordingly if the correct ERF is used. Therefore, there is an urgent need to develop ERFs for the CNOSSOS-EU exposure estimation method.

## 5. Conclusions

The equal distribution method, as used in the CNOSSOS-EU for population assignment, reduces the number of people exposed to traffic noise above 55 dB L_den_ and 50 dB L_night_. If the old ERFs were to be used with this new exposure data, the estimated number of highly annoyed and highly sleep disturbed citizens would decrease accordingly. However, the estimated adverse health effects may stay at the same level or even increase if the ERFs derived for the CNOSSOS-EU exposure estimation method were to be used. Therefore, new ERFs will be needed for the CNOSSOS-EU when applied to the health impact assessment of transportation noise. 

## Figures and Tables

**Table 1:** The effect of population assignment method on the estimated number of people exposed to road traffic noise in Helsinki in 2016. In both methods, the calculations are based on façade noise levels modeled with CNOSSOS-EU [10].

	Old Method	New Method
L_den_ (dB)		
55–59	124,800	85,800
60–64	118,500	50,700
65–69	51,800	18,400
70–74	25,600	8200
>75	900	100
L_night_ (dB)		
50–55	130,500	59,300
55–59	52,000	20,400
60–64	28,900	9600
65–69	2200	400
>70	0	0

**Table 2:** Characteristics of study groups used for determination of the exposure–response functions.

	Road Traffic Noise	Rail Traffic Noise
Number of respondents	6754 (HAV and HSDV)	3331 (HAV)/3303 (HSDV)
Aged 30–69 years (%)	88	71
Female (%)	58	58
Sensitive to noise (%)	26	26

**Table 3:** Community tolerance levels for road traffic noise.

	BUILDING	MAX20	WINDOWS
L_ct_den_	86.1 dB (r = 0. 7703)	83.7 dB (r = 0. 9517)	80.3 dB (r = 0.9529)
L_ct_night_	87.3 dB (r = 0.7907)	87.1 dB (r = 0.9443)	82.1 dB (r = 0.9305)

**Table 4:** Number of individuals highly annoyed and/or highly sleep disturbed because of road traffic noise in Helsinki in 2017.

	Exposure Estimation Method × ERF		
Outcome	BUILDING × BUILDING	EQUAL × BUILDING	EQUAL × WINDOW
HA_v_	13,100	5000	13,700
HSD_v_	11,300	4400	8200

× Sign for multiplication (number of exposed times percentage of highly annoyed/sleep disturbed).

## Data Availability

The data are not publicly available for privacy and ethical reasons (as defined by GDPR). The data presented in this study are available on request from the corresponding author in a restricted form.

## Supplementary Materials

The following are available online at https://www.mdpi.com/article/10.3390/ijerph19031314/s1. Partial translation of the Environmental Health Survey Questionnaire; Table S1. Number of respondents exposed to MAX20 below 45 dB L_den_ or 40 dB L_night_; number of highly annoyed or highly sleep disturbed respondents in this group (N_highly); percentage of highly annoyed (HA_v_) and percentage of highly sleep disturbed (HSD_v_) respondents.
