# Supplementary material for: Exposure–Response Functions for the Effects of Traffic Noise on Self-Reported Annoyance and Sleep Disturbance in Finland: Effect of Exposure Estimation Method
Source: Int J Environ Res Public Health. 2022 Jan 25;19(3):1314. doi: 10.3390/ijerph19031314 (PMC8834923; doi:10.3390/ijerph19031314)
Supplement: Supplementary file 1 [file ijerph-19-01314-s001.zip › ijerph-1459712-supplementary.pdf]

Exposure-Response Functions for the Effects of Traffic Noise on Self-Reported Annoyance and Sleep Disturbance in Finland: Effect of Exposure Estimation Method

Tarja Yli-Tuomi, Anu W. Turunen, Pekka Tiittanen and Timo Lanki

Supplementary Material

Partial translation of the Environmental Health Survey Questionnaire

Questions concerning background information; dwelling and living environment; and environmental noise have been translated from Finnish to English for supplemental information of Yli-Tuomi et al. 2022, IJERPH xx

BACKGROUND INFORMATION

1. Date of birth (dd.mm.yyyy)

2. Gender

3. Marital status

4. Do you have children?

5. Are there any children less than 18 years old living in your household?

6. Education

7. Present work status

8. The closest occupational group that you belong to.

9. Your working hours.

10. Average monthly income of your household before taxes (=gross earnings).

DWELLING AND LIVING ENVIRONMENT

11. How satisfied you are with your living environment?

12. What type of dwelling do you live in?

1 Owner-occupied dwelling
2 Rental dwelling
3 Part-ownership/right-of-occupancy dwelling
4 Assisted living building/rehabilitation home/old-age home
5 Another type

13. What type of building do you live in?

1 Multi-story residential building

2 Balcony access block/row house/two-family house

3 Single family house

14. When was your home building constructed?

1 Before 1900

2 1900–1940

3 1941–1960

4 1961–1980

5 1981–2000

6 In 2001 or after

15. Which type of ventilation system is in your dwelling?

1 Natural ventilation

2 Exhaust ventilation system (mechanical exhaust)

3 Balanced ventilation system (mechanical supply and exhaust)

4 I do not know

16. How long have you lived in your current home?

1 Less than one year

2 1–3 years

3 4–9 years

4 10–19 years

5 20 years or longer

17. The floor you are living on.
First floor is the floor on the ground level. If you are living at an apartment with several floors, please select the floor where your bedroom is located.

1 Basement

2 1st floor, ground level

3 2nd floor

4 3rd floor

5 4th floor

6 5th-7th floor

7 8th floor or higher

18. Orientation of the windows in your home

1 Facing the street

2 Facing the yard

3 Facing both the street and the yard

19. Where are your bedroom's windows facing?

1 Facing the street
2 Facing the yard

20. Orientation of the balcony in your home?

1 Facing the street

2 Facing the yard

3 Facing both the street and the yard

4 No balcony

21. Is the yard you usually use facing the street?

1 No
2 Yes
3 I don't use any yard

22. Do you usually keep your bedroom window open while sleeping during the warm season (May – September)?

1 No
2 Yes

23. What kind of pets do you have?

1 Cat
2 Dog
3 Bird
4 Rodent
5 Reptile
6 Other animal(s)
7 No pets

24. How satisfied you are with the following conditions in your living environment?

Green areas include forests, parks, fields, natural grasslands, wetlands and bare rocks.

	Very unhappy	Fairly unhappy	Not happy, nor unhappy	Fairly happy	Very happy
Public transportation	1	2	3	4	5
Services	1	2	3	4	5
Safety	1	2	3	4	5
Cleanliness	1	2	3	4	5
Buildings' condition	1	2	3	4	5
Neighbors	1	2	3	4	5
Amount of green areas	1	2	3	4	5
Quality of green areas	1	2	3	4	5

25. Do you stay regularly at a secondary residence (owned by you, rented, otherwise)?

26. How much time do you usually spend at a secondary residence during the warm season (May-September)?

27. How much time do you usually spend at a secondary residence during the cold season (October - April)?

ENVIRONMENTAL NOISE

Based on the next questions, your exposure to different noise sources and possible disadvantages caused by the exposure will be estimated.

28. To what extent are you exposed to the following noises at home?

	Not at all	Slightly	Moderately	Very	Extremely
a) Road traffic noise	1	2	3	4	5
b) Rail traffic noise	1	2	3	4	5
c) Tram traffic noise	1	2	3	4	5
d) Air traffic noise	1	2	3	4	5
e) Construction and maintenance work noise	1	2	3	4	5
f) Noise caused by next-door neighbors (speaking, music, etc.)	1	2	3	4	5
g) Other neighborhood noise (schools, sports fields, restaurants, etc.)	1	2	3	4	5

29. Are you usually disturbed by the following noises (i.e. noise annoys you, disturbs your concentration, etc.) indoors at home when the windows are closed?

	Not at all	Slightly	Moderately	Very	Extremely
a) Road traffic noise	1	2	3	4	5
b) Rail traffic noise	1	2	3	4	5
c) Tram traffic noise	1	2	3	4	5
d) Air traffic noise	1	2	3	4	5
e) Construction and maintenance work noise	1	2	3	4	5
f) Noise caused by next-door neighbors (speaking, music, etc.)	1	2	3	4	5
g) Other neighborhood noise (schools, sports fields, restaurants, etc.)	1	2	3	4	5

30. Are you usually disturbed by the following noises (i.e. noise annoys you, disturbs your concentration, etc.) when you are at home in the yard or at the balcony?

	Not at all	Slightly	Moderately	Very	Extremely
a) Road traffic noise	1	2	3	4	5
b) Rail traffic noise	1	2	3	4	5
c) Tram traffic noise	1	2	3	4	5
d) Air traffic noise	1	2	3	4	5
e) Construction and maintenance work noise	1	2	3	4	5
f) Noise caused by next-door neighbors (speaking, music, etc.)	1	2	3	4	5
g) Other neighborhood noise (schools, sports fields, restaurants, etc.)	1	2	3	4	5

31. Is your sleep usually disturbed by the following noises (i.e. noise prevents you from falling asleep, wakes you up) at home?

	Not at all	Slightly	Moderately	Very	Extremely
a) Road traffic noise	1	2	3	4	5
b) Rail traffic noise	1	2	3	4	5
c) Tram traffic noise	1	2	3	4	5
d) Air traffic noise	1	2	3	4	5
e) Construction and maintenance work noise	1	2	3	4	5
f) Noise caused by next-door neighbors (speaking, music, etc.)	1	2	3	4	5
g) Other neighborhood noise (schools, sports fields, restaurants, etc.)	1	2	3	4	5

32. Are you usually disturbed by the following noises at your working or studing places?

	Not at all	Slightly	Moderately	Very	Extremely
a) Road traffic noise	1	2	3	4	5
b) Rail traffic noise	1	2	3	4	5
c) Tram traffic noise	1	2	3	4	5
d) Air traffic noise	1	2	3	4	5
e) Construction and maintenance work noise	1	2	3	4	5
f) Noise from machines or industrial processes	1	2	3	4	5
g) Noise caused by human beings (speech, moving obstacles)	1	2	3	4	5
h) Music	1	2	3	4	5

33. Has traffic noise influenced on the amount of time you usually spend in your yard or on balcony?

1 No
2 Yes, some
3 Yes, a lot
4 I don’t have a yard or balcony

34. Has traffic noise in your living environment affected on the amount of time you keep your windows open?

1 No
2 Yes, some
3 Yes, a lot

35. Did traffic noise have an influence when you selected the present living environment?

1 No, I didn’t consider traffic noise as an important factor
2 No, the number of options was limited
3 Yes, some
4 Yes, a lot

36. What is your opinion on the following statements that describes you?

		Strongly agree	Agree	Neutral	Disagree	Strongly disagree
a)	No one should care if someone is playing his/her stereos at full volume.	1	2	3	4	5
b)	Noise wakes me up easily.	1	2	3	4	5
c)	I resent if my neighbors are noisy.	1	2	3	4	5
d)	I get used to most of the noise without any special difficulties.	1	2	3	4	5
e)	Sometimes noise gets on my nerves and gets me irritated.	1	2	3	4	5
f)	Even my favorite music disturbs me when I try to concentrate.	1	2	3	4	5
g)	It is hard for me to relax if it’s noisy.	1	2	3	4	5
h)	It’s easy for me to concentrate whatever happens around me.	1	2	3	4	5
i)	I get irritated with people who are so noisy that I’m not able to fall asleep or to get my work done.	1	2	3	4	5
j)	Living by a noisy road does not disturb me if the dwelling is cozy.	1	2	3	4	5
k)	I am sensitive to noise.	1	2	3	4	5

AIR POLLUTION ***Questions 37 - 50***

TRANSPORTATION AND GREEN AREAS ***Questions 51 – 67***

RISK PERCEPTIONS AND ATTITUDES ***Questions 68 - 74***

HEALTH AND WELFARE ***Questions 75 - 85***

SMOKING AND ALCOHOL USE ***Questions 86 - 93***

Table S1. Number of respondents exposed to MAX20 below 45 dB L_{den} or 40 dB L_{night}; number of highly annoyed or highly sleep disturbed respondents in this group (N_highly); percentage highly annoyed (HA_v) and percentage highly sleep disturbed (HSD_v) respondents.

	N_total	N_highly	HA_v or HSD_v
Road traffic noise			
L_{den} < 45 dB	680	2	0.3%
L_{night} < 40 dB	1632	14	0.9%
Rail traffic noise			
L_{den} < 45 dB	2494	1	0.0%
L_{night} < 40 dB	2742	3	0.1%
